# Regional differences, distribution dynamics and convergence of multi-dimensional grain security level in major grain producing areas in China

**DOI:** 10.1371/journal.pone.0339498

**Published:** 2026-03-11

**Authors:** Jing Cheng, Jiaqi Guo

**Affiliations:** School of Humanities and Social Sciences, Jiangsu University of Science and Technology, Zhenjiang, Jiangsu Province, China; Macau University of Science and Technology, MACAO

## Abstract

Based on panel data from China’s 13 major grain – producing provinces between 2014 and 2023, this study constructs a multidimensional grain security evaluation index system, incorporating the dimensions of quantity, quality, ecology, and capacity. Using the entropy method, kernel density estimation, Dagum Gini coefficient, and convergence model, we analyze the regional differences, dynamic evolution, and spatial convergence of grain security levels. The results show that: (1) grain security level is generally increasing; there are obvious spatial differences in grain security level among; (2) There are obvious spatial differences in grain security levels among regions, showing the trend of “Northeast> central> eastern> western”; (3) α Convergence analysis shows that the central region converges, while the northeast, east and west regions diverge; both absolute β convergence and conditional β convergence exist in the whole region and each region. Compared with previous studies, this paper provides new insights into the dynamic evolution, spatial distribution and regional convergence of multidimensional grain security in China’s major grain producing areas by integrating a multi-dimensional framework including quantity, quality, ecology and capacity. The research has enriched the evaluation system of grain security and laid a solid foundation for policy optimization.

## 1. Introduction

Grain security is an important cornerstone of national security. It is related to national development, people’s well-being and social stability. Ensuring grain security is a top priority for the government. In order to ensure grain security, the scientific distribution of grain production areas is crucial. Scientific layout of grain production areas is also crucial. Since the 18th National Congress of the Communist Party of China, China has made remarkable achievements in the field of grain security, with the comprehensive grain production capacity steadily improving, and the grain security guarantee system gradually improved. The Communist Party of China said in the report to the 20th National Congress:“ Strengthening the foundation of grain security on all fronts”, “Ensure that China’s stable grain supply is firmly held in its own hands”, “Ensure that the security of grain, energy and resources, and important industrial and supply chains”. The report stressed that through policy support, science and technology -enabled, regional coordination and governance system construction, the mechanism to guarantee the income of grain farmers and the compensation mechanism for the interests of major grain producing areas should be improved to ensure that major grain producing areas have enthusiasm for grain production, providing important guidance for the long-term development of China’s grain security.

To further consolidate the foundation of grain security, China will implement a new round of actions to increase grain production capacity by 100 million tons in 2024 to promote a steady and increased production rate in grain production. However, in recent years, China has faced an increasingly severe situation at home and abroad. Due to multiple factors such as geopolitics, trade friction, global climate and natural disasters, China’s grain production and agricultural trade are limited. On the one hand, grain supply and demand have been in a tight balance for a long time, and structural shortage problems remain prominent. In particular, we still need to rely on imports of feed grains and oil crops and other important agricultural products. On the other hand, agricultural science and technology support capacity needs to be further improved, the low rate of conversion of scientific and technological achievements, insufficient talent support and other problems still exist.

In addition, there are differences in grain security capacity among regions, and grain production in some regions still faces the dual pressure of natural conditions and weak infrastructure. With this background, it is particularly important to study the multi-dimensional grain security issues in China’s main grain producing areas in depth. This is not only an inevitable requirement for the implementation of the national grain security strategy, but also a key initiative to optimize the regional grain production layout and enhance the ability to guarantee grain security. A systematic analysis of the multidimensional level of grain security in major grain-producing regions can provide a scientific basis for policy formulation and help to achieve coordinated regional development and a dynamic balance of grain security. Through a systematic analysis of the multi-dimensional grain security level of major grain producing areas, it can provide scientific basis for policy formulation and help realize the dynamic balance of coordinated development of regions and grain security.

Based on the relevant studies of current scholars, scholars have made theoretical analysis on the connotation and realization path of grain security in major grain producing areas, and empirical research has been carried out in terms of indicator system construction and measurement. First, the theoretical analysis of the main grain producing areas. Scholars discuss the policy mechanism and sustainable development path of major grain producing areas from multiple perspectives. Chen and Lu (2025) [1] discussed the spatial and temporal trend of agricultural green production level in major grain producing areas, and put forward useful insights for promoting green and low-carbon agricultural development in major grain producing areas through inter-provincial cooperation, attracting foreign investment, promoting the integration of rural industries and increasing farmers’ income. Cheng et al.(2017) [2] used the geographic detector model to identify the dominant factors affecting the spatial-temporal coupling of grain-economy-ecosystem in major producing areas, providing theoretical support for optimizing the land use structure and improving the land carrying capacity. In terms of the agricultural structure, LU et al.(2013) [3] put forward an interval-probability of agricultural structure optimization model (IPAPSOM), used to deal with such as grain security policy constraints, rural household income growth and ecological environment protection uncertainty factors, provides a variety of decision makers, help them determine the ideal agricultural structure optimization strategy. In terms of grain production, Wen yuan et al. (2022) [4] found that by expanding the planting area, improving the level of mechanization and increasing the financial transfer payment, the average grain yield per unit area could increase by 27.5%, which significantly increased the grain output. Weijuan (2023) [5] built contains resistance, resilience and reconstruction ability of agricultural development toughness evaluation system, points out that the main grain producing areas in “grain ecology” dual security target of technology innovation, investment and mechanized cultivated land resources is the key factor to improve agricultural toughness, to enhance the ability to cope with risk provides the theoretical basis. In the sustainable development of agriculture, some scholars believe that the sustainable development mechanism can effectively promote the benign development of Major Grain Producing Areas.For example, Ederson Diniz Ebling et al. (2025) [6] found that reasonable agricultural management models, such as no-till forage and grain production pairing, can protect soil, reduce soil erosion, and ensure grain production at the same time, reflecting the dual positive impact of sustainable development mechanism on ecological environment and grain yield. Wanxu C et al. (2023) [7] focused on the problem of global grain production and spatial-temporal mismatch of cultivated land, suggesting that sustainable development measures such as optimizing resource allocation could alleviate the mismatch and improve the overall efficiency of grain production areas. O.R. L et al. (2022) [8] emphasized in the study of dryland wheat production that it is not easy to achieve sustained high yield and quality due to the constraints of natural conditions such as climate and soil, as well as technical and economic factors, reflecting that various factors in grain production are interwoven with each other, which may lead to deviations between actual effects and expectations.

Second, the research on the connotation of grain security. The connotation of grain security is deepening with the development stage and external environment, and the academic circle is gradually shifting from a single output orientation to a comprehensive and dynamic perspective. Chen et al. (2024) [9] studied the grain security risks in the Yangtze River Delta region of China, analyzed the association between water shortages, grain production and grain trade, and emphasized the important impact of water resources on grain security. Yang and Xu (2024) [10] starting from the coupling and coordination relationship between digital economy and green agriculture development, discussed the promoting effect of digital economy development on grain security, pointing out that the development of digital economy can effectively improve the efficiency and sustainability of agricultural production. Fang et al. (2022) [11] analyzed the spatial changes in the total primary productivity of cultivated land in China and its challenges to sustainable grain production and grain security, and pointed out that the improvement of cultivated land productivity is crucial to ensure grain security. In terms of grain quantity security, H L S (2016) [12] explored the intervention measures of researchers and farmers to improve crop yields in rural China, demonstrated the potential of localized methods for global grain production increase, and proposed suggestions on how international cooperation can be used to jointly address grain security challenges. JG et al. (2010) [13] discussed the dual impact of perennial grains on grain security and ecosystem security from the perspective of ecosystem, and proposed methods to improve grain yield and ecosystem stability by planting perennial grains. Pahl-Wostl C (2019) [14] points out that the governance of the water-energy-grain security nexus faces multi-level coordination challenges, emphasizing the importance of effective coordinated management of this complex system. This provides a new perspective for a deeper understanding of the interconnected governance of grain security with water, energy, and other elements. Barrett (2010) [15] further stresses the dynamic adaptability of indicator systems, advocating for adjusting weights based on regional characteristics to reflect the diverse needs of grain security. Alam B F M et al. (2024) [16] explored the ripple effect of disruptions in the grain supply chain from a new perspective on supply chain resilience, analyzing the factors that enhance this effect. This approach broadens the scope of grain security, moving beyond mere production and supply volumes to consider the impact of coordination and stability across all supply chain stages on grain security. Kadiri et al. (2025) [17] extend the concept of food security beyond calorie sufficiency to climate-shock resilience, showing that climate-resilient growth stabilizes crop income and supply chains, thereby improving availability, access and stability dimensions in low- and middle-income countries. Long et al. (2025) [18] reframe food security as “sunlight-harvest efficiency,” demonstrating that bioengineering photosynthesis to raise light-to-biomass conversion in field crops can generate more edible calories per unit land and input, offering a resource-sparing route to stable, climate-resilient food availability. These studies provide a multi-dimensional perspective for deeply understanding the connotation and realization path of grain security, and also provide a theoretical basis for the formulation of scientific and reasonable grain security policies.

The third is the research on the construction of the grain security level evaluation system. Scholars have gradually formed a research paradigm of “multi-dimensional collaboration and dynamic iteration”. Cheng and Yin (2024) [19] selected 17 indicators covering climatic conditions and human activities to assess the grain security in northern China at the level of grain production and found that the level of grain production is one of the key factors affecting grain security. Godfray et al. (2010) [20] discussed the grain security challenges of how to feed 9 billion people in 2050 from a global perspective, and proposed a multi-dimensional grain security evaluation system, including grain production, distribution and consumption. Pinstrup-Andersen (2009) [21] defines grain security and discusses its measurements, emphasizing the multi-dimensional characteristics of grain security, including the availability, access, utilization and stability of grain. Guo Shumin et al. (2007) [22] Based on grey correlation analysis of production links, key factors such as effective irrigation area and disaster area were screened out, providing methodological support for connotation quantitative research. Zhao Huijie and Yu Fawen (2019) [23] introduced ecological indicators such as pesticide use and agricultural film residue to promote the transformation of the evaluation system to green. Han Yang (2022) [24] Starting from the development stage theory, he advocated that short-term supply and demand contradictions and medium-and long-term resource constraints should be included in the evaluation framework, emphasizing that the system should evolve dynamically with internal and external challenges. At present, it is generally agreed that grain security evaluation needs to coordinate four dimensions of quantity security, quality security, ecological security and health security [25]. For example, the quality of grain can be evaluated through nutrient analysis, the circulation resilience can be measured by road density, and the consumption potential can be reflected through per capita disposable income, so as to construct a comprehensive evaluation framework that takes into account both sustainability and inclusiveness. Shokhrukh-Mirzo J et al. (2022) [26] took Bangladesh as an example to discuss the strategies and evaluation system to improve grain security from the perspective of cost control, and found that controlling the cost of grain security is the key, but different cost control strategies have different effects. Zabala A (2018) [27] emphasized the fundamental position of land resources in the grain security evaluation system, pointed out that the sustainable use of land resources has a profound impact on grain security, and relevant indicators should be better integrated into the evaluation system. Dai et al. (2025) [28] introduce a spatial-equilibrium index into the food-security evaluation system by linking county-level supply–demand gaps with optimal cropping zones simulated with the PLUS model, enabling dynamic iteration across quantity, regional match and governance scenarios. Wang & Tong (2025) [29] establish a five-dimensional index system for Shanxi Province and apply entropy-TOPSIS to assess food security during 2001–2022, demonstrating that economic security and resource sustainability weights increase over time and offering a provincial-level paradigm that integrates policy and ecological dimensions into dynamic evaluation.

To sum up, domestic and foreign scholars have made great achievements on the grain security level in major grain producing areas, which also provides a rich literature basis for this study, but there is still room for expansion in some aspects. First of all, the existing research focuses on the traditional indicators such as grain output and cultivated land area in the selection of indicators, but the comprehensive consideration of multidimensional grain security level (such as quality, stability and market) is still insufficient. Secondly, most of the existing studies focus on the regional differences and dynamic evolution of grain yield or cultivated land utilization efficiency, while less attention is paid to the future convergence characteristics of the grain security level in major grain producing areas, so it is difficult to fully reflect the grain security level in major grain producing areas. In addition, in terms of system construction and index selection of grain security level, the research mainly focuses on macro analysis at the national or regional level, and there is a lack of targeted analysis of major grain producing areas. In 2001, China officially divided 13 major grain-producing areas, 7 main sales areas and 11 basic balanced areas. By 2003,13 provinces (autonomous regions) including Heilongjiang, Jilin, Liaoning, Hebei and Henan were further identified as major grain-producing areas. These main producing areas occupy a key position in China’s grain production pattern and become the core force to stabilize grain supply. In view of this, this paper selects the representative major grain producing areas, based on the 13 provinces from 2014–2023, constructs the evaluation index system of grain security level from quantity security, quality security, ecological security and ability security, analyzes the regional difference, distribution dynamics and convergence of grain security level using entropy method, kerenel nuclear density estimation method, dagum gini coefficient and convergence model.

Compared with the existing literature, the possible marginal contribution of this paper is that: First, the innovative expansion of theoretical frameworks. Breaking through the limitations of traditional grain security research that focuses solely on yield and arable land, this approach is based on a four-dimensional framework of “quantitative safety, quality safety, ecological safety, and capability safety.” It constructs a multi-dimensional indicator system tailored to the development characteristics of major grain-producing areas, revealing the synergistic driving mechanisms of agricultural technological innovation, infrastructure upgrades, and the alleviation of ecological constraints on the overall level of grain security. Second, the deepening and integration of the methodological level. Through the coupling analysis of entropy empowerment and kerenel kernel density estimation method and Dagum gini coefficient decomposition, the regional differences and spatial distribution characteristics of the contribution of economic development gradient, resource endowment heterogeneity and policy implementation efficiency to the dynamic distribution and difference are first clarified. Meanwhile, combined with the α convergence and β convergence model, the dynamic characteristics of “late advantage” and “path dependence” in central and western China and east are revealed, providing a new perspective for regional collaborative policy design. Third, the precise breakthrough of policy implications. By optimizing the agricultural structure and regional layout, and the “Party and government responsibility-market coordination” mechanism, it provides a direct basis for the formulation of differentiated transfer payment standards, priority division of science and technology promotion, and dynamic adjustment of arable land protection red line in the implementation rules of the grain Security Law.

The first part of the article is the introduction; The second part is about the study design; The third part is the dynamic analysis of measurement and distribution; The fourth part is divided into regional differences and source analysis; The fifth part is the convergence analysis; The sixth part is divided into a discussion; The seventh part is the conclusion and policy recommendations; The eighth section is for ref.

## 2. Research design

### 2.1. Indicator construction

In order to comprehensively and accurately evaluate the grain security system, meet the needs of the people, maintain the ecological balance and the ability to resist risks, the grain security level of the 13 major grain producing areas is comprehensively considered based on the theoretical logic, historical logic and practical logic of grain security. This paper will select 16 indicators from the four dimensions of quantity security, quality security, ecological security and ability security.

Malthus emphasized the tension between population growth and grain supply in his population theory, highlighting the importance of ensuring adequate grain supply. Therefore, when considering grain quantity security, grain output, grain output per unit area and grain production per capita are the key factors. Besides, Jin (2025) [30] mentioned the total output value of agriculture, forestry, animal husbandry and fishery when studying the quality of rural living environment in the old revolutionary base area of Dabie Mountain. This index reflects the production situation of various agricultural products including grain production, as well as the overall level and scale of agricultural production in a region.

High-quality grain varieties are often rich in higher levels of protein, vitamins and minerals and other nutrients, and the proportion of their planting directly affects the nutritional quality of grain supply. The determination of the use of chemical fertilizers and pesticides in the process of grain production is based on the scientific method of nutrient composition analysis. The core function of fertilizers is to provide mineral elements such as nitrogen, phosphorus, and potassium, which directly determine the synthesis levels of micronutrients including protein in grains, vitamin B complex, zinc, and iron. Agricultural diesel serves as the sole fuel source for field operations (deep plowing, straw incorporation, precision spraying). Its consumption indirectly reflects “plowing precision” and “homogeneous straw shredding,” with the latter influencing the mineralization rate of soil organic matter and ultimately determining the available concentrations of trace elements. In constructing the evaluation index of the dimension of quality safety, the use of pesticide, the amount of chemical fertilizer and the use of agricultural diesel are the primary factors [31]. In addition, meat is an important source of high-quality protein, fat, vitamins (such as B vitamins) and minerals (such as iron, zinc, etc.). The increase in total meat production can provide residents with more nutrients and help meet the human body’s demand for a variety of nutrients, especially for ensuring an adequate supply of high-quality protein. The selection of these indicators is designed to ensure that grain not only meets the demand in quantity, but also provides the variety of nutrients needed by the human body qualitatively to meet the challenges of malnutrition and related health problems.

Ecological security focuses on the security of grain production to the carrying capacity of resources and environment, including resource consumption and environmental impact. The evaluation of effective irrigated area is based on the principle of soil ecology. Irrigation is a necessary part of grain production, and the water consumption for irrigation directly affects the growth and quality of grain crops. In exploring the relationship between the membership of the Water User Association (WUA) and grain security, Suraj M M (2025) [32] suggests that the participation of the Water User Association helps to improve grain security conditions.

However, selecting the amount of agricultural plastic film used and the affected area as the secondary index is based on the research results on pollution emission and ecological impact in environmental science The increase in the area affected by natural disasters (such as floods, droughts, typhoons, pests and diseases) or man-made disasters (such as industrial pollution, deforestation, etc.) means that the ecosystem is more vulnerable to natural disasters (such as floods, droughts, typhoons, pests and diseases) or man-made disasters. The destruction of this structure will inevitably affect the function of the ecosystem, threaten the ecological security, and then have a certain impact on the grain security.

Ability security involves grain production, circulation, consumption and sustainable development capacity. Per capita disposable income determines consumption power and investment possibility; As a market signal, the agricultural production price index affects producers’ decisions according to the price theory, and then affects grain supply and the overall ability of the industry; Road density reflects the situation of transportation infrastructure; From the perspective of logistics transportation, the higher the guarantee of agricultural transportation and grain circulation; The total power of agricultural machinery reflects the level of agricultural mechanization. Based on the theory of technological progress, its growth improves production efficiency, alleviates the impact of labor transfer, enhances the stability of agricultural production and the ability to resist risks, which is the technical and equipment guarantee of capacity safety.

### 2.2. Research method

#### 2.2.1. Entropy value method.

In this paper, the data of 16 indicators selected from 13 provinces of major grain producing areas from 2014 to 2023 were taken as research samples and analyzed by entropy method. According to the overall impact of the numerical change of each index, the empowerment of each index is calculated and the weight is determined. This can avoid the influence of subjective factors and improve the objectivity and accuracy of the evaluation results. The specific calculation steps are performed as follows:

(1) The data were standardized to make it comparable.

$${\text{For positive indicators},\text{ r}}_{\text{ij}}=\frac{{\text{a}}_{\text{ij}}-\text{min}({\text{a}}_{\text{ij}})}{\text{max}({\text{a}}_{\text{ij}})-\text{min}({\text{a}}_{\text{ij}})}$$ (1)

$$\text{For negative indicators},\;{\text{r}}_{\text{ij}}=\frac{\text{max}({\text{a}}_{\text{ij}})-{\text{a}}_{\text{ij}}}{\text{max}({\text{a}}_{\text{ij}})-\text{min }({\text{a}}_{\text{ij}})}$$ (2)

(2) Calculate the proportion of each evaluation index.

$${\text{P}}_{\text{ij}}=\frac{{\text{r}}_{\text{ij}}}{\sum_{\text{i}=\text{1}}^{\text{n}}{\text{r}}_{\text{ij}}}$$ (3)

(3) Computing the information entropy of each index.

$${\text{E}}_{\text{j}}=—\text{k}\sum_{\text{i}=\text{1}}^{\text{n}}{\text{p}}_{\text{ij}}\text{ln}{\text{p}}_{\text{ij}},\text{among i}=\text{1},\text{2},\text{ n},\text{ k}=\frac{\text{1}}{\text{ln}\text{n}}$$ (4)

(4) The utility value of the information entropy of each indicator was calculated.

$${\text{G}}_{\text{j}}=\text{1}-{\text{E}}_{\text{j}}$$ (5)

(5) Calculate the index weights.

$${\text{W}}_{\text{j}}=\frac{{\text{G}}_{\text{j}}}{\sum_{\text{j}=\text{1}}^{\text{m}}{\text{G}}_{\text{j}}}$$ (6)

(6) Calculate the grain security composite score.

$${\text{S}}_{\text{i}}=\sum_{\text{j}=\text{1}}^{\text{m}}{\text{W}}_{\text{j}}{\text{r}}_{\text{ij}}$$ (7)

Where m represents the number of indicators of the grain security evaluation system, r_ij_ is the standard value of j indicators in year i, and a_ij_ represents the original value of j indicators in year i.

#### 2.2.2. Kernel density estimation.

Nuclear density estimation is a non-parametric method for estimating the probability density function of random variables. It has great flexibility and adaptability when dealing with complex, multi-peak or unknown distribution data. This paper uses the kernel density estimation method to analyze the spatial distribution dynamics of the multi-dimensional grain security level. The calculation formula is as follows:


$$\text{f }(\text{x})=\frac{\text{1}}{\text{nh}}\sum_{\text{n}=\text{1}}^{\text{n}}\text{k}(\frac{{\text{x}}_{\text{i}}-\text{X}}{\text{h}})$$ (8)

$$\text{k}=\frac{\text{1}}{\sqrt{\text{2}\pi \text{e}}}e^{-\frac{x^{\text{2}}}{\text{2}}}$$ (9)

Where k (x) represents the kernel function; n represents the number of observations; x_i_ represents the independently distributed observations; X represents the average; and h represents the kernel width.

#### 2.2.3. The Dagum Gini coefficient.

The Dagum Gini coefficient is a commonly used measure of inequality used to measure the degree of inequality in a set of data or distribution. The Dagum Gini coefficient can be decomposed into intra-group coefficient, inter-group coefficient and super variation density coefficient, that is Dagum=Within the group Gw+ interblock Gb+Super density Gt. The calculation formula is as follows:


$$\begin{array}{c} G=\frac{\sum_{j=\text{1}}^{k}\sum_{h=\text{1}}^{k}\sum_{i=\text{1}}^{n_{j}}\sum_{r=\text{1}}^{n_{h}}\left|y_{ij}-y_{hr}\right|}{\text{2}n^{\text{2}}\overset{―}{y}} \end{array}$$ (10)

Where, G represents the overall Gini coefficient, k represents the number of regions, n represents the number of provinces, y_ij_ (y_hr_) represents the comprehensive evaluation score of each province within the region j (h), and $\overset{―}{y}$ represents the average of the multi-dimensional grain security index of China as a whole.

In this paper, the main grain producing areas are divided into four regions: eastern, central, western and northeast. Hebei, Shandong and Jiangsu are located in the eastern region; Henan, Hubei, Hunan, Anhui and Jiangxi are located in the central region; Inner Mongolia and Sichuan are located in the western region of China; Heilongjiang, Jilin and Liaoning provinces are located in northeast China. According to the decomposition method of Dagum Gini coefficient, Gw within the group reflects the internal level gap within each region respectively, indicating the contribution of the difference of grain security index in the four regions of eastern, central, western and northeast; The group of Gb reflects the horizontal gap between regions and indicates the contribution of differences between the four regions; The hypervariable density Gt reflects the overlap phenomenon in each region, indicating the contribution of the overlap between samples to the overall difference. In this paper, it mainly refers to the increase in the overall difference caused by the occurrence of low provinces within high levels of grain security and high provinces with low levels of grain security. The Dagum Gini coefficient makes up for the problem of measuring regional gap because other methods cannot solve the problem of overlapping data, and can better identify the source of regional gap.

#### 2.2.4. Convergence analysis.

Existing literature demonstrates that convergence tests across regions or industries have evolved into a landscape of “multiple methodologies coexisting”. Bilgili & Ulucak (2018) [33] conducted a G20 ecological footprint study employing deterministic, stochastic, and Phillips-Sul club convergence methods to identify common means, stationary differences, and endogenous clubs. The OECD Energy Technology Budget paper (2022) [34] prioritized panel β convergence and α club methods to quantify “catch-up speed of less developed countries”. European Oil Price Analysis (2014) [35] utilized SUR-ADF and club methods to determine whether industry-household price differentials formed subgroups. While these three approaches each have distinct advantages, their applicability varies significantly in “small cross-sections—short panels” scenarios. Yuting X et.al. (2023) [36] and Guozhu L et.al. (2023) [37] first use α-convergence to confirm the narrowing of regional gaps in green innovation and ecological resilience, and then apply β-convergence to quantify the catch-up speed and half-life, providing measurable evidence for regional convergence potential. Fousekis (2007) [38] further employed β-convergence to gauge the catch-up speed and half-life of state-level agricultural TFP across the United States.

By comparison, in order to explore the convergence characteristics of grain security levels in major grain producing areas, α and β convergence analysis were used to determine whether regional differences tend to converge or diverge. First, α convergence analysis was used to explore whether regional differences in multidimensional grain security levels tend to converge or diverge. Then, the β convergence analysis was used to predict whether areas with lower grain security levels could catch up with higher safety levels at a faster rate. Moreover, conditional variables can be seamlessly introduced to provide marginal effect ranking for differentiated policies. The α and β convergence analysis is detailed as follows.

(1) α convergence

α Convergence theory refers to the decreasing trend of the deviation of the multidimensional grain security level between different regions over time. By calculating the coefficient of variation of the degree of grain security, whether the convergence of the grain security level can be judged. If the coefficient of variation decreases year by year, it indicates that the difference in grain security level between different regions is gradually narrowing, that is, there is α convergence at this time. In this study, the coefficient of variation is used to describe the convergence of α, as follows:


$$\begin{array}{c} \sigma =\frac{\sqrt{\frac{\sum_{i=\text{1}}^{n}{\left(D_{i,t}-\overset{―}{D_{i,t}}\right)}^{\text{2}}}{n}}}{\overset{―}{D_{i,t}}} \end{array}$$ (11)

Where i represents different provinces and t represents the year, D_i, t_ represents the multi-dimensional grain security index of province i in the year t.

(2) β convergence

β convergence theory analyzes whether regions with low multidimensional grain security levels can catch up with those with high grain security levels at a faster rate over time, thus gradually narrowing the regional differences. Ultimately, areas with both high and low levels of grain security will achieve convergent levels of growth. This convergence phenomenon can further be divided into two forms: absolute β convergence and conditional β convergence. Absolute β convergence means that the final level of multidimensional grain security will converge to the same steady-state level in all regions, regardless of the initial gap in the grain security level. Conditional β convergence means that the grain security level of each region will show a trend of convergence under the premise of controlling the factors affecting the multidimensional grain security level. This means that despite differences in multidimensional grain security levels across regions, all converge to similar steady-state levels. With D_i, t_ indicates the degree of multidimensional grain security in i in t, D _i, t + 1_ indicates the degree of multidimensional grain security in t + 1, µ _i_, ν_t_ and ε_t_ represent spatial fixed effects, temporal fixed effects and random disturbance terms respectively. The computational models of absolute β convergence and conditional β convergence are as follows:


$$\begin{array}{c} \begin{array}{c} \text{ln}\left(\frac{{\text{D}}_{\text{i},\text{t}+\text{1}}}{{\text{D}}_{\text{i},\text{t}}}\right)=\alpha +\beta \;\text{ln}\;{\text{D}}_{\text{i},\text{t}}+\mu_{\text{i}}+\nu_{\text{t}}+\epsilon_{\text{it}} \end{array} \end{array}$$ (12)

$$\begin{array}{c} \begin{array}{c} \text{ln}\left(\frac{{\text{D}}_{\text{i},\text{t}+\text{1}}}{{\text{D}}_{\text{i},\text{t}}}\right)=\alpha +\beta \;\text{ln}\;{\text{D}}_{\text{i},\text{t}}+\lambda \sum_{\text{j}=\text{1}}^{\text{n}}{\text{Control}}_{\text{i},\text{t}}+\mu_{\text{i}}+\nu_{\text{t}}+\epsilon_{\text{it}} \end{array} \end{array}$$ (13)

α is the constant term, and β is the convergence coefficient. If β is significantly less than 0, it indicates that the multidimensional grain security level has the convergence characteristic; otherwise, it has the dispersion characteristic. The convergence rate can be calculated by − ln (1 + β)/ T. λ is the coefficient of the control variable, Control _i, t_ represents the control variables affecting the degree of multidimensional grain security, including urbanization rate (UR), financial support (FS), economic development level (EDL) and industrial structure heightening (ISS). Among them, the urbanization rate reflects the degree of population transfer from rural areas to urban areas, which affects grain demand and agricultural production mode. Its calculation formula is the proportion of urban population in the total population. The level of financial support is measured by the ratio of general budget expenditure to gross domestic product (GDP), which reflects the government’s investment in promoting economic development and ensuring people’s livelihood, and has an important impact on grain security. The level of economic development is measured by per capita GDP, which reflects the average economic welfare and purchasing power of residents and has a direct impact on grain security. The calculation formula of high industrial structure is the proportion of added value of the primary industry in GDP multiplied by 1, plus the proportion of added value of the secondary industry in GDP multiplied by 2, plus the proportion of added value of the tertiary industry in GDP multiplied by 3. This index reflects the optimization and upgrading degree of industrial structure by assigning corresponding weights to different industries, which in turn affects the level of grain security.

### 2.3. Variable description and data source

In this study, the balance panel data of 13 provinces (autonomous regions and municipalities) included in the major grain producing areas from 2014 to 2023 were selected. These data are mainly derived from China Statistical Yearbook, China Agricultural Yearbook, China Water Resources Statistical Yearbook and some local statistical yearbooks. For missing data in some years, interpolation was used in this study.

## 3. Measurement and distribution dynamics

### 3.1. Measure results

Based on the theoretical framework of grain security, a multi-dimensional grain security evaluation index system including quantity security, quality security, ecological security and capacity security was constructed for major grain producing areas, and the entropy value method was used to objectively assign weights to 16 indicators. The results are shown in Table 1, Quantity security (43.47%) contributed the most to the overall level, followed by ecological security (22.77%), ability security (19.92%) and quality security (16.73%). The weights of various dimensions show significant hierarchical differences, revealing the security pattern of major grain producing areas and showing the hierarchical characteristics of “Capacity leading-ecological capacity coordination”: The core position of quantity security (weight 43.47%) indicates that Policies in major producing areas focus on ensuring the basic capacity of grain production, to maintain a high supply level through large-scale production and technological innovation, it is highly in line with the national strategic orientation of “grain storage on the ground and grain storage in technology”; The binding role of ecological security (weight 22.77%) highlights the rigid requirements of agricultural production environmental sustainability, reflects the practical response of major producing areas in the fields of cultivated land protection and non-point source pollution control, and verifies the symbiotic logic of grain security and environmental protection under the guidance of “ecology first”; The synergistic value of quality and ability safety (total weight 36.65%) reflects the gradual emphasis of policies on quality safety and risk prevention and control. Among them, quality safety (16.73%) points to the upgrading demand of residents’ dietary structure, while ability safety (19.92%) covers the long-term guarantee mechanism such as infrastructure resilience and market regulation efficiency. This weight structure shows that in the past ten years, China’s grain security strategy in major grain producing areas has shifted from a single capacity expansion to a diversified and coordinated mode of “stabilizing capacity, consolidating ecology and improving capacity”, which provides empirical evidence for optimizing the combination of grain security policy tools.

**Table 1:** Multidimensional grain security evaluation system.

Dimension	Index	Unit	Indicator nature	Weight
Quantitative security	Grain yield	Ten thousand tons	Positive	7.36%
	Grain output per unit area	Kg/ha	Positive	2.12%
	Per capita grain production	kg/ person	Positive	25.03%
	Total output value of agriculture, forestry, animal husbandry and fishery	100 million	Positive	6.06%
Quality security	Pesticide use	ton	Negative	2.90%
	The amount of fertilizer applied	Ten thousand tons	Negative	2.34%
	Agricultural diesel consumption	Ten thousand tons	Negative	1.29%
	Total meat production	Ten thousand tons	Positive	10.20%
Ecological security	Waterlogging area	thousand hectares	Positive	11.67%
	Effective irrigation area	thousand hectares	Positive	7.49%
	Affected area	thousand hectares	Negative	1.58%
	Usage amount of agricultural plastic film	ton	Negative	2.03%
Capacity security	Road intensity	Kilm/ square kilometer	Positive	6.16%
	Agricultural product production price index	–	Negative	4.14%
	Total power of agricultural machinery	Ten thousand kilowatts	Positive	2.86%
	Per capita disposable income of residents	yuan	Positive	6.76%

From the level of specific indicators, the two indicators of grain per capita output and waterlogged area occupy a high weight in the system, which indicates that the per capita grain ownership and the ability of drainage and reclamation in agricultural cultivation play a vital role in ensuring grain security. According to the data of the third National Land Survey released in 2021, China’s total arable land area is about 1.918 billion mu, and the per capita arable land area is only 1.36 mu. To firmly guard the red line of arable land protection is to ensure the red line of 1.8 billion mu of arable land. However, there is an interaction between grain production and disaster prevention capacity, and the perfect agricultural infrastructure construction and drainage reclamation capacity are the key factors to ensure the stability of grain production. Therefore, governments at all levels actively implement the responsibility system for farmland protection and grain security assessment, formulate clear production targets, make every effort to achieve a good harvest of grain, promote the stability of animal husbandry and the progress of modern fisheries, and jointly consolidate the bottom line of national grain security.

The entropy value method is used to analyze the multidimensional comprehensive level of grain security in 13 major grain producing areas in China from 2014 to 2023. As shown in Table 2, the analysis results show the following characteristics: ①In terms of time trend, the comprehensive score of multi-dimensional grain security in major grain producing areas from 2014 to 2023 showed an overall upward trend, with the growth rate of 2023 being about 32.25% compared with 2014, and exceeding 5.0 in 2020. Among them, the lowest growth rate of Hubei province was 23.72%, and that of Inner Mongolia was 52.92%. On the one hand, the major grain producing provinces are all major agricultural provinces in China, and the grain self-sufficiency rate in most provinces exceeds 100%. The Heilongjiang province is more than six times higher. The improvement of grain security and the increase in grain transfer from major producing areas have fundamentally ensured national grain security. On the other hand, since the implementation of the policy in major grain producing areas, agricultural production capacity has been continuously enhanced and the grain industry economy has developed steadily. It has promoted the increase of grain output, the improvement of agricultural infrastructure and the structural reform of the agricultural supply side, and promoted the continuous growth of the multidimensional comprehensive index of grain security in major grain producing areas of China.

**Table 2:** Grain security index.

	2014	2015	2016	2017	2018	2019	2020	2021	2022	2023
Hebei	0.2831	0.2815	0.3006	0.3202	0.3371	0.3467	0.3649	0.3746	0.3755	0.3722
Nei Monggo	0.2699	0.2669	0.2592	0.2958	0.3353	0.3554	0.3733	0.3913	0.4120	0.4128
Liaoning	0.2260	0.2455	0.2594	0.2580	0.2654	0.2944	0.2940	0.3215	0.3212	0.3212
Jilin	0.3241	0.3317	0.3282	0.3524	0.3414	0.3686	0.3946	0.4181	0.4172	0.4182
Heilongjiang	0.4881	0.4951	0.4698	0.5576	0.5644	0.5817	0.6468	0.6803	0.6752	0.6772
Jiangsu	0.3785	0.3915	0.3980	0.4258	0.4440	0.4621	0.4647	0.4743	0.4653	0.4713
Anhui	0.3262	0.3382	0.3378	0.3718	0.3780	0.4009	0.4218	0.4289	0.4392	0.4394
Jiangxi	0.2139	0.2222	0.2256	0.2266	0.2318	0.2625	0.2675	0.2680	0.2741	0.2735
Shandong	0.4148	0.4242	0.4424	0.4781	0.4890	0.4937	0.5083	0.5326	0.5414	0.5583
Henan	0.3946	0.4180	0.4241	0.4272	0.4446	0.4695	0.4802	0.4710	0.4937	0.4901
Hubei	0.2741	0.2872	0.2902	0.3092	0.3144	0.3277	0.3348	0.3509	0.3592	0.3613
Hunan	0.2684	0.2806	0.2815	0.2801	0.2838	0.3098	0.3300	0.3175	0.3359	0.3320
Sichuan	0.2737	0.2869	0.2987	0.2893	0.3005	0.3141	0.3379	0.3304	0.3413	0.3416
Ensemble	0.3181	0.3284	0.3319	0.3532	0.3638	0.3836	0.4014	0.4123	0.4193	0.4207

②In terms of spatial trend, the comprehensive level of multidimensional grain security in Heilongjiang, Shandong, Henan and Jiangsu is relatively high, which are 5.84,4.88,4.51 and 4.38 respectively; Sichuan, Hunan, Liaoning and Jiangxi were relatively low, with 3.11,3.02,2.81 and 2.47 respectively. Heilongjiang had the highest level of grain security, about 2.36 times that of Jiangxi province. The reasons may include: Heilongjiang, known as the “granary of China,” boasts abundant arable land resources, with its total grain production, commercial volume, and export volume ranking first in the country for consecutive years; Shandong’s grain industry started early and transformed quickly, leveraging geographical advantages to provide excellent conditions for grain industry distribution and logistics radiation; Henan, as an old agricultural powerhouse, maintains stable production capacity; Jiangsu, with superior climate conditions, has high production potential. However, the relatively low grain security level in Sichuan, Hunan, Liaoning and Jiangxi may be due to the differences in agricultural science and technology level, industrial structure and natural condition endowment. The high level of grain security is not only due to its unique natural conditions and strong production capacity, but also closely related to the local positive investment and efforts in ecological protection, nutrition and health. In general, there are significant spatial differences in grain security levels in China’s main grain producing areas.

**Fig 1:**
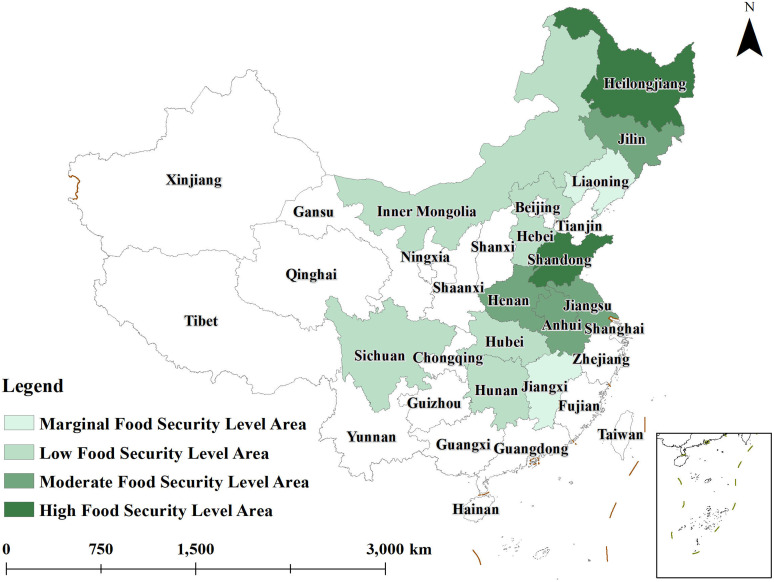
Distribution of grain security levels in 2014.

**Fig 2:**
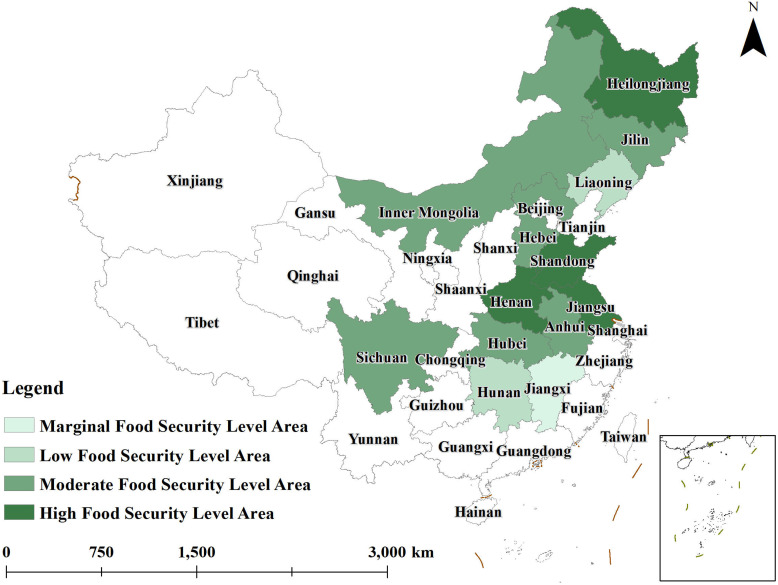
Distribution of grain security levels in 2018.

**Fig 3:**
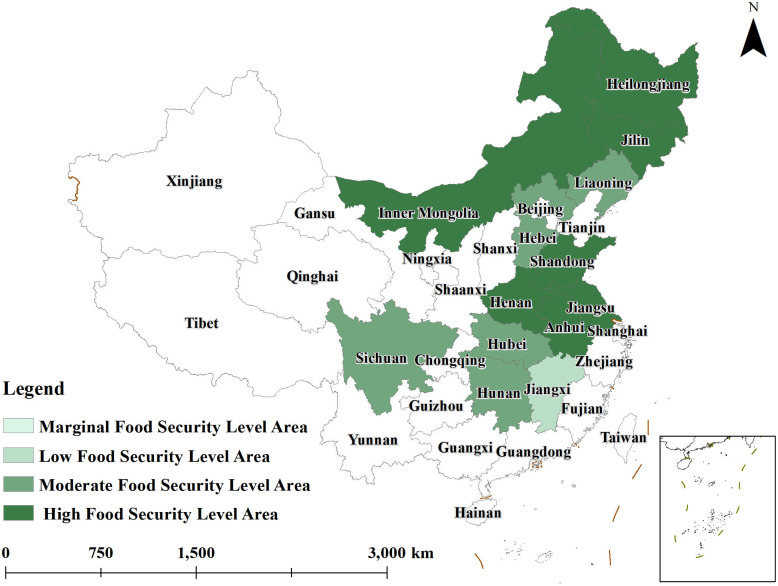
Distribution of grain security levels in 2023.

To gain a more intuitive understanding of the spatial evolution of grain security levels in major grain-producing regions from 2014 to 2023, ArcGIS10.8 software was used to create distribution maps of grain security levels for the years 2014,2018, and 2023. The agricultural resilience of these regions was categorized into four levels using a grading method: 0.000–0.250 as the lowest level, 0.250–0.300 as the low level, 0.300–0.400 as the medium level, and 0.400–0.700 as the high level. These classifications are illustrated in Figs 1–3.

As shown in Fig 1, the overall grain security level in major grain-producing regions was relatively low in 2014. Liaoning and Jilin were in the lowest grain security zones; Hunan, Hubei, Inner Mongolia, Hebei, and Sichuan were in the low grain security zones; Henan, Anhui, Jiangsu, and Jilin were in the medium grain security zones, while Shandong and Heilongjiang were in the high grain security zones. During this period, the grain security situation in major grain-producing regions was relatively weak.

By 2018 (Fig 2), the number of provinces with high grain security levels increased to include Jiangsu and Henan. Sichuan, Hubei, Inner Mongolia, and Hebei were upgraded from low to medium grain security levels. Liaoning also moved from a lower to a medium grain security level, while Jiangxi remained in the lower grain security category. During this period, all major grain-producing provinces saw improvements in their grain security levels, but the scope of high grain security areas remained limited.

By 2023 (Fig 3), the grain security level of major grain-producing areas has significantly improved. Inner Mongolia, Jilin and Anhui have significantly improved their comprehensive grain security scores, rising from medium to high grain security levels; Liaoning and Hunan have risen to medium grain security levels; and only Jiangxi province remains in the low grain security level area. Most of the provinces with a significant increase in the comprehensive score of grain security are China’s old agricultural provinces, which not only have geographical advantages in agricultural production, but also take active measures such as strengthening agricultural technology promotion, optimizing agricultural planting structure and improving agricultural infrastructure construction to improve grain production capacity and ensure grain security.

### 3.2. Distributed dynamics

**Fig 4:**
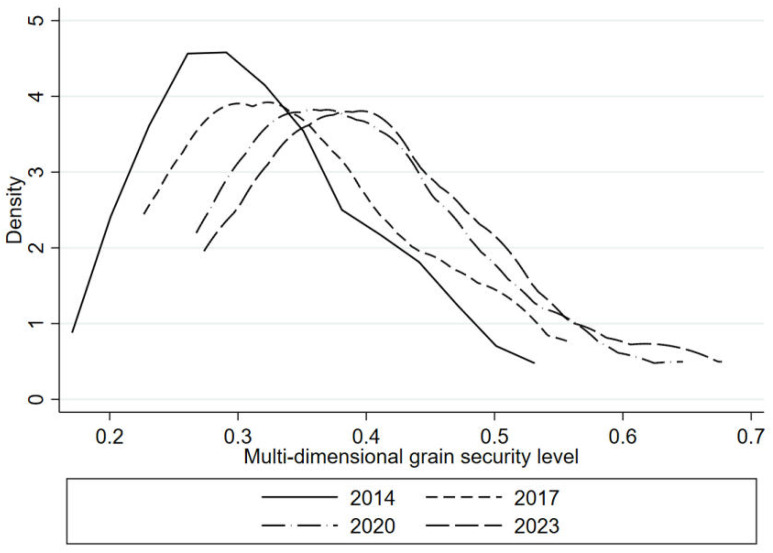
The dynamic evolution of multidimensional grain security.

Kernel density estimation is a non-parametric statistical method used to estimate the probability density function of a random variable. It “smooths” the sample data and reveals the distribution form of the data. It is especially suitable for unknown distribution forms or complex data distribution. In this study, four years, namely 2014,2017,2020 and 2023, were selected to measure the spatial distribution and dynamic evolution of multidimensional grain security level in China’s main grain producing areas by using kernel density estimation method, as shown in Fig 4.

From the distribution characteristics of the nuclear density curve, it can be seen that with the passage of time, China’s multidimensional grain security level presents a positive development trend. Specifically, the overall nuclear density curve moves to the right, which intuitively reflects the steady improvement of grain security level in the past decade. From the gradual decrease of the peak of the nuclear density curve, the difference of multi-dimensional grain security level in China’s main grain producing areas is gradually increasing. The increase of this difference may be related to the unbalanced development of natural conditions, policy support, technological progress and other aspects of the major producing areas. For example, some major producing areas may have made significant progress in agricultural science and technology input and infrastructure construction, thus promoting the rapid improvement of grain security level, while other regions are relatively lagging behind. In general, despite regional differences, China’s grain security level in major grain producing areas has made significant progress in the past decade, providing a strong guarantee for national grain security.

## 4. Regional differences and source analysis

In order to further explore the regional differences and sources of multidimensional grain security in China’s main grain producing areas from 2014 to 2023,13 main grain producing areas were divided into four regions: eastern, central, western and northeastern regions according to the relevant national geographical standards, and the Dagum Gini coefficient and its decomposition method were used for comparative analysis. Table 3 shows the results of the regional differences and their dynamic evolution, with the following characteristics: (1) The Gini coefficient of grain security level in China’s main grain producing areas increased from 0.133 in 2014 to 0.136 in 2023, showing a fluctuating increase in the overall difference, and showing a fluctuating trend of “upward-downward-upward-downward”. This indicates that the overall difference degree has been expanded. (2) The regional differences are relatively stable, while the inter-regional differences fluctuate significantly. The inter-group Gini coefficient fluctuates within the range of 0.058 ± 0.0095, and its changing trend is consistent with the overall difference. However, the intra-group Gini coefficient fluctuates only by 0.003, indicating that the inter-regional differences are the main driving force for the evolution of the overall difference. (3) The decomposition of the dagum Gini coefficient shows: the highest contribution rate is between groups, followed by the super variable density effect, and the lowest contribution rate within groups. The average values are 42.93%,34.12%, and 22.94%, respectively. This indicates that the main reason for the imbalance and inadequacy in China’s major grain-producing areas is the heterogeneity of resource endowments and policy implementation efficiency across regions, followed by the impact of inter-regional interactions, while the contribution of intra-regional differences is relatively limited. The above findings show that although the provinces in the main grain producing areas have achieved the improvement of grain security level by relying on their own resource advantages, the trend of regional development concentration has intensified the spatial imbalance, highlighting the necessity of optimizing regional coordination mechanism and differentiated policy design.

**Table 3:** Results of Dagum Gini coefficient and contribution rate.

Year	Gini coefficient				Contribution rate (%)		
	Ensemble	Within the group	interblock	Super dense	Within the group	interblock	Super dense
2014	0.133	0.03	0.056	0.046	22.799%	42.337%	34.865%
2015	0.131	0.031	0.054	0.046	23.482%	41.155%	35.364%
2016	0.125	0.029	0.058	0.038	23.485%	46.457%	30.058%
2017	0.143	0.032	0.067	0.044	22.284%	46.933%	30.783%
2018	0.141	0.032	0.062	0.047	22.871%	43.761%	33.368%
2019	0.128	0.03	0.055	0.044	23.063%	42.720%	34.216%
2020	0.134	0.03	0.052	0.051	22.736%	39.258%	38.006%
2021	0.137	0.031	0.064	0.043	22.266%	46.429%	31.305%
2022	0.133	0.031	0.053	0.049	23.294%	39.994%	36.712%
2023	0.136	0.031	0.055	0.05	23.147%	40.250%	36.603%

**Fig 5:**
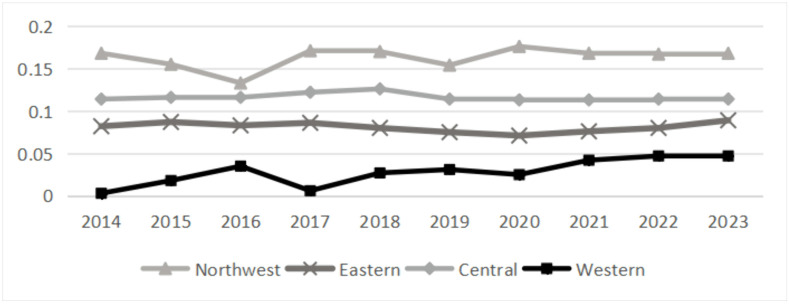
Gini coefficient within group.

Based on the above analysis, the dynamic characteristics of regional differences are further investigated. As shown in Fig 5, the Gini coefficient in the eastern and western region groups remained basically unchanged from 2014 to 2023, while the gini coefficient in the northeast region group showed a fluctuation trend, while the gini coefficient increased in the middle range of fluctuation. Among them, the gini coefficient in the northeast group has the highest differentiation, and the average Gini coefficient is 0.163. It can be seen that the gap in the grain security level in the northeast group is the largest, which showed the characteristics of the first falling and then rising, and rebounded after declining again in 2019. The central region (0.116) and the eastern region (0.081) followed, while the western region had the lowest value of 0.028. The comparative analysis shows that the regional differences of multi-dimensional grain security level in China’s main grain producing areas are “Northeast> central> eastern> western”.

## 5. Convergence analysis

### 5.1. α convergence test

**Fig 6:**
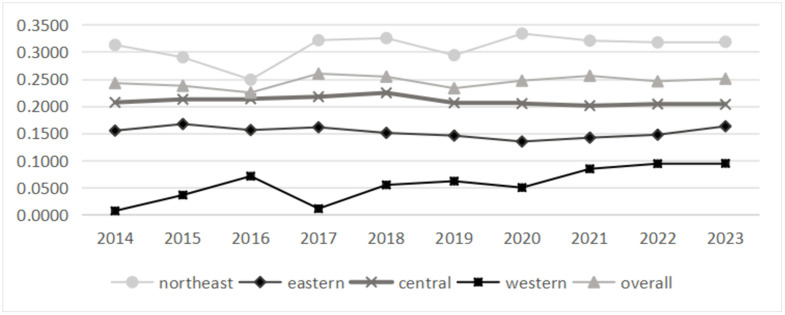
The trend of coefficient of variation.

According to the α convergence formula, the evolution and convergence trend of the coefficient of variation of grain security level in major grain producing areas are calculated, as shown in Fig 6. On the whole, the coefficient of variation of multidimensional grain security in China’s main grain producing areas shows a rising trend of “decline-rise-decline-rise”, and the fluctuation is relatively flat. In 2014–2023, the coefficient of variation increased by 3.35%, as no convergence. The coefficient of variation in the northeast, eastern and western regions all increased, but not convergence. Only the central region showed a downward trend, which was a converging phenomenon. It can be concluded that there are α convergence characteristics in central China, and the difference of grain security level decreases; The northeast, eastern and western regions showed a divergent trend, indicating that the regional grain security level was significantly unbalanced, which was consistent with the results of the Gini coefficient mentioned above. It can be seen that regional differences are more and more obvious in the development of nearly ten years, which is closely related to the differences in natural, economic and industrial structure within the region. For example, Heilongjiang province in northeast China has prominent agricultural advantages, while Liaoning and Jilin provinces have limited agricultural input and insufficient coordination, which leads to the inconsistency between regional development speed and the improvement of grain security level, and aggravates the imbalance. The eastern region is economically developed and urbanization is advancing rapidly. Different provinces attach different importance to agriculture and adopt different strategies. Industrialization and urbanization in some provinces lead to the occupation of farmland and loss of labor force. Despite the advantages of science and technology and capital, it is difficult to ensure the planting area and labor force stability. However, the natural conditions in western China are complex, and the differences in terrain, climate and water resources among provinces are large. For example, Inner Mongolia is rich in agricultural and animal husbandry resources, but its infrastructure and technology promotion are weak; Sichuan has many mountains and faces difficulties in large-scale production and modernization; its economy lags behind, fiscal input is limited, and there is a lack of coordination mechanism, which leads to significant differences in grain security levels and aggravates with time.

### 5.2. β convergence test

#### 5.2.3. Absolute β convergence analysis.

Table 4 reports the absolute convergence of β for grain security levels in major grain producing areas. It can be seen that after controlling for the fixed effects of years and cities, the overall, northeast, eastern, central and western regions of China’s grain security level β convergence is all negative. In addition, the main grain producing areas passed 5%, the northeast region passed 10%, the central region failed the significance test, and the central and western regions passed 1%. This means that the overall level of China’s main grain producing areas and the four major grain producing areas have an absolute β convergence trend of grain security. If other potential influencing factors and variables are ignored, those regions with lower performance in multidimensional grain security levels are expected to achieve faster development through the “catch-up effect”. That is to say, with the implementation of grain security policies in major grain producing areas and the implementation of supply-side reform of agricultural structure, the level of grain security in all regions will tend to a common stable state.

**Table 4:** β absolute convergence.

Variable	Ensemble	Northeast	East	Midland	West
Convergence coefficient	−0.414** (−2.98)	−0.912* (−3.36)	−0.288 (−2.59)	−0.578*** (−4.61)	−0.173*** (−5.7)
constant term	−0.453** (−2.87)	−0.972* (−3.16)	−0.282 (−2.12)	−0.673** (−4.32)	−0.207 (−3.07)
Area fixed effect	Yes	Yes	Yes	Yes	Yes
Time fixed effect	Yes	Yes	Yes	Yes	Yes
R^2^	0.3835	0.7263	0.6704	0.6725	0.4023

Note: The values in parentheses are the t-value of the significance test; the upper symbols * * *, * * and * represent the tested values as significant at the levels of 1%, 5% and 10%, respectively. Table 5 is the same.

#### 5.2.3. Condition β convergence analysis.

Table 5 reports the β convergence results for grain security in China’s main grain producing areas. After adding urbanization rate (UR), financial support (FS), economic development level (EDL) and industrial structure heightening (ISS) as control variables, as well as controlling the fixed effect of cities and years, it can be seen that the β condition convergence of grain security level in China’s main grain producing areas is negative, indicating that there is β condition convergence. Among them, China’s grain production area and central region passed the 1% significance test, while northeast China passed the 5% significance test, and eastern and western regions did not pass the test. That is to say, the growth rate of the northeast, east, central and western regions will be affected by their institutional and structural factors, and converge to their respective steady states. The steady state of each economy is different, and if the economic system and structural factors remain unchanged, the gap will not disappear.

**Table 5:** β conditional convergence.

Variable	Ensemble	Northeast	East	Midland	West
Convergence coefficient	−0.655*** (−6.67)	−1.216** (−7.93)	−0.423 (−0.7)	−0.647*** (−6.03)	−1.041 (−1.7)
lnUR	−0.666 (−1.23)	1.750234 (0.49)	0.140 (0.09)	−1.111 (−1.68)	−3.774 (−0.72)
lnFS	−0.210 (−0.63)	−0.0289 (−0.09)	0.0280 (0.07)	−0.407 (−0.31)	0.554 (0.08)
lnEDL	−1.74 (−1.97)	−7.70 (−1.38)	−1.34 (−0.07)	−1.10 (−0.40)	−0.00001 (−0.62)
lnISS	−0.590 (−2.80)	−0.926 (−2.88)	0.802 (0.77)	0.282 (0.93)	−1.928 (−0.71)
constant term	1.109 (1.66)	0.0161 (0.01)	−2.383 (−0.80)	−0.728 (−0.64)	5.409 (1.25)
Area fixed effect	Yes	Yes	Yes	Yes	Yes
Time fixed effect	Yes	Yes	Yes	Yes	Yes
R^2^	0.4579	0.8109	0.7709	0.6977	0.7364

## 6. Discuss

China has a vast territory, and the main grain producing areas have significant differences in natural conditions, economic development and agricultural infrastructure, which leads to the imbalance of grain security level among regions. Although China has made great achievements in the field of grain security, there are still many challenges to achieve a higher quality and more balanced grain security system, so it is important to study. In view of the limitations of data and perspective, this paper has some shortcomings in research. In the future, it can be expanded from the following aspects:

(1) Expand global vision and multidisciplinary integration: grain security is the core issue of global stability and development. Future research should put domestic grain security under a global perspective and comprehensively consider the impact of external factors such as international trade and climate change. In the subsequent research, we will gradually make the global factor into an “exogenous shock pool” to study the marginal effect of price-trade shock on provincial convergence speed. At the same time, we should strengthen interdisciplinary integration, draw on research methods and theories from biology, geography, environmental science, economics and other disciplines, and fully reveal the internal laws and influencing mechanisms of grain security.By strengthening international cooperation, establishing data sharing mechanisms, and standardizing data collection standards and methods, we can effectively integrate theories and methods from different disciplines and obtain comprehensive, accurate, and comparable data on a global scale. For example, by cooperating with international organizations and research institutions in other countries to participate in the construction of the Global Grain Security Database project and share data resources. In the future, we can also use coupling and other methods to calculate the general equilibrium model to realize the two-way feedback between “global and regional”.
(2) Refined research scale and integration of emerging technologies: The current research is mainly based on provincial data, and in the future, it can be further refined to the city, county and even village level. Combined with high-resolution remote sensing data, geographic information system (GIS) technology and field investigation, the spatial distribution dynamics and evolution trend of grain security level can be accurately analyzed. At the same time, we will actively explore the convergence points between emerging technologies such as big data, artificial intelligence and the Internet of Things and grain security research, develop innovative research methods and tools, and provide more efficient and accurate technical support for grain security.This can be solved by exploring various channels for data acquisition, establishing a mechanism for data sharing and cooperation, and applying for research funding and special funds. At the same time, strengthening policy support and guidance to promote industry-university-research cooperation will also help ensure the effective application and promotion of emerging technologies in grain security research.
(3) Focus on social and economic factors and strengthen policy coordination: grain security is closely related to social and economic factors. In the future, more attention should be paid to the structure of rural labor force, farmers ‘income level, the degree of agricultural industrialization and consumers’ eating habits. At the same time, it is necessary to further strengthen the research on the coordination of grain security policies. Future research should integrate the “dual control of carbon and food” mechanism into the coordinated framework of food security policies, systematically analyzing the interaction and transmission pathways between carbon emission constraints and yield-increasing incentives, analyze the interrelationship and mechanism of action among different policies. The general equilibrium can be calculated by dynamic multi-agent coupling analysis, establish a policy coordination evaluation model, simulate the impact of different policy combinations on grain security, and provide scientific basis for optimizing the policy system and improving the efficiency of policy implementation. Only by normalizing the multi-subject, multi-objective and multi-tool policy coordination evaluation can we truly embed “food security” into the overall framework of high-quality economic and social development and avoid the one-sided governance of “food security”.

## 7. Conclusion and policy proposal

### 7.1. Conclusion

This paper makes an in-depth analysis of the regional differences, distribution dynamics and convergence of multi-dimensional grain security level in China’s main grain producing areas from 2014 to 2023, and draws the following conclusions:

(1) From 2014 to 2023, the multidimensional comprehensive level of grain security in China’s main grain-producing areas showed an overall positive trend of improvement. This is mainly attributed to the high regard for grain security by the state and the continuous advancement of agricultural infrastructure construction. During this period, the achievements of agricultural science and technology innovation were widely applied in the main grain-producing areas. Increased investment in farmland water conservancy facilities and road transportation effectively improved agricultural production conditions, reduced the impact of natural risks on grain production, and enhanced the disaster resistance of grain production. As a result, it strongly promoted the steady growth of grain yields and advanced the multidimensional comprehensive level of grain security.
(2) The regional differences in multidimensional grain security levels in China’s main grain-producing areas generally show a fluctuating upward trend, indicating that development remains uneven and insufficient. The disparity follows the pattern of “Northeast> Central> Eastern> Western,” reflecting a concentration of development across regions and exacerbating spatial imbalances. This urgently requires optimizing regional coordination mechanisms and differentiated policy designs to promote balanced and sustainable grain security development.
(3) Convergence analysis shows that the central region is converging, with a trend of narrowing differences in grain security levels; the northeastern, eastern, and western regions have not converged. At the same time, there is an overall and regional trend of absolute β convergence in major grain-producing areas, indicating that low-level regions have the potential to achieve faster development through a “catch-up effect.” Conditional convergence analysis of β shows that there is a conditional β convergence in the northeastern, eastern, central, and western regions as well as in major grain-producing areas, suggesting that when considering internal regional differences and external conditions, each economy converges to its own steady state.

We recognize that these conclusions may not be universally applicable to grain security studies in different countries and regions. Due to significant differences in natural conditions, socioal economic environments, policy frameworks, and cultural backgrounds, the specific manifestations and influencing factors of grain security issues can vary. For example, dietary culture and consumption habits in different regions can influence the types and quantities of grain demand; different agricultural production methods can affect grain security levels; variations in land systems and social security policies can impact the efficiency of grain production and distribution; international market factors, such as fluctuations in grain prices and changes in trade policies, can also influence grain security.

### 7.2. Policy proposal

(1) Improving policy support for grain security

Taking into account regional differences, characteristics of production links and characteristics of grain crops, targeted policies should be formulated to enhance policy coordination and avoid conflicts and disconnection. For example, differentiated support should be implemented for major producing areas and major sales areas, and subsidies, price support and insurance policies should be coordinated. Improvement of the mechanism for guaranteeing the income of grain farmers and the mechanism for compensating the interests of the main producing areas. At the same time, we should adjust policies in a timely manner according to the changes in domestic and foreign situations, pay attention to market dynamics and technological changes, and ensure that policies meet the needs of grain production and development. In addition, we will actively innovate policy means, introduce market and social forces, carry out pilot production trusteeship services, set up industrial development funds, promote diversified development of the grain industry, and enhance market competitiveness and risk resistance.

(2) Optimizing China’s agricultural structure and regional layout

We need to optimize the agricultural structure according to local resource endowments, develop competitive industries in accordance with local conditions, rationally adjust the proportion of planting, animal husbandry and fishery, and improve the efficiency of resource utilization. For example, develop corresponding industries according to water resources conditions, promote the combination of planting and breeding; promote regional specialization layout, build characteristic industry belts and clusters; strengthen regional coordination and cooperation, establish a mechanism for connecting production and marketing, and promote the circulation of agricultural products. At the same time, we should closely combine market demand to optimize agricultural structure, strengthen market monitoring and information services, guide production to adapt to consumption upgrading, develop high-quality, green and characteristic agriculture, strengthen brand building, and enhance the added value of agricultural products and market competitiveness.

(3) Full implementation of party and government responsibility for grain security

Party committees and governments at all levels should define their responsibilities, refine them to departments and posts, coordinate them with the party committee, and the government should be responsible for implementing supervision. Local targets and tasks should be formulated in accordance with national strategies and incorporated into performance assessment. We will establish and improve supervision, assessment and accountability mechanisms, formulate scientific evaluation standards, reward the good and punish the bad, and ensure that responsibilities are fulfilled. We should also strengthen coordination and cooperation, establish cross-departmental coordination mechanisms, jointly solve problems and form synergy. At the same time, we should strengthen publicity and education, raise public awareness, encourage public participation in supervision, and create a good atmosphere for the whole society to jointly guarantee grain security.

## Supporting information

S1 Data Data. (XLSX)
